# A current view on the involvement of protein phosphatase PP2A subunits in the responses of plants to abiotic stress at the subcellular level

**DOI:** 10.1007/s00299-026-03931-5

**Published:** 2026-08-20

**Authors:** Csaba Máthé, Márta M-Hamvas, Tamás Garda, Zoltán Kónya

**Affiliations:** 1 Plant Cell and Developmental Biology Research Group, Faculty of Science and Technology, University of Debrecen, Debrecen, Hungary; 2 Department of Medical Chemistry, Faculty of Medicine, University of Debrecen, Egyetem Ter 1, 4032 Debrecen, Hungary

**Keywords:** Protein phosphatase PP2A, Abiotic stress tolerance, Drought, Salt, Heat/cold stress, Chromatin, Cytoskeleton, Membrane compartments

## Abstract

PP2A is the most abundant member of the non-metal-dependent serine-threonine phosphatases in eukaryotes. It is an important player in the reversible phosphorylation of proteins and involved in a wide variety of cellular processes, from signal transduction events to cell cycle regulation and a large number of metabolic processes. At the subcellular level, there is a comprehensive knowledge on PP2A-mediated regulation of chromatin and cytoskeletal organization and there is increasing evidence for its relevant functions in the organization of membrane compartments. Relatively little is known about the related regulatory functions of this enzyme complex in the responses of plants to abiotic stresses. However, recent research has revealed several aspects of these relationships at the subcellular level, mainly for osmotic/salt and heat stress. PP2A is a family of three-subunit holoenzyme complexes, where “A” are scaffolding, “B” are regulatory and “C” are catalytic subunits. Here we discuss the involvement of all these subunits in the regulation of stress-related changes in chromatin, cytoskeleton (microtubule and microfilament), endomembrane (including ER) and plastid/mitochondrial organization and emphasize how they sustain the functioning of plant cells under multiple abiotic stresses. There are crucial subunits in this respect, for example A1, C3 and C4 as well as “B” regulatory subunits such as FASS and members of the B and B’ subfamilies. PP2A is involved in stress hormone (featuring ABA) signaling at multiple levels. In light of recent research, we show that different PP2A holoenzymes link together diverse stress-related signaling mechanisms.

**Supplementary Information:**

The online version contains supplementary material available at https://doi.org/10.1007/s00299-026-03931-5.

## Introduction

### PP2A and its functions in plants

PP2A belongs to the PPP (phosphoprotein phosphatase) family of serine-threonine protein phosphatases and represents the most abundant member of this family in most eukaryotes (Shi 2009). The holoenzyme consists of three distinct subunits: “A” (scaffolding), “B” (regulatory) and “C” (catalytic) (Farkas et al. 2007; Shi 2009). The presence of multiple isoforms of these subunits gives a high number of combinations in the holoenzyme, allowing the wide range of substrates subject to dephosphorylation as well as its variability in subcellular localization (Shi 2009; Virshup and Shenolikar 2009).

The scaffolding (A) subunits are responsible for the structural integrity of the holoenzyme. In Arabidopsis there are three members of this family and among them, A1 (RCN, Roots Curl in Naphthyltphtalamic acid) is the most studied and has not only a structural role, but also a crucial one in axial organ regulation, especially root development (Blakeslee et al. 2008). Rice has only one “A” subunit (Yu et al. 2021).

The “B” subunit family shows the highest structural and functional variability among the three, to such an extent, that even their molecular weights span a wide range (54–130 kDa). Their total number varies between species: in Arabidopsis, for instance, 17 of these subunits are known to date (Farkas et al. 2007; Spinner et al. 2013; Máthé et al. 2023). They are grouped into three subfamilies: B, B’ and B”, with multiple members within each subfamily. Animal cells contain the B’” subfamily as well, but this is absent in plants (Bheri et al. 2020). The roles of “B” subunits depend on the type of isoform, but in general, they assure the subcellular localization, the substrate specificity and/or even the activity of a given holoenzyme form (Virshup and Shenolikar 2009; Máthé et al. 2023). We mention here two examples that are relevant for the present review. The B’γ subunit regulates the functionality of the holoenzyme under multiple stress conditions (Konert et al. 2015a; Durian et al. 2020; Máthé et al. 2023). FASS (TON2) is a member of the B” subunit family. It binds a specific holoenzyme form (A1 + FASS + C3/C4) to a larger protein complex to localize holoenzyme to the microtubules (MTs) – both at the level of cortical microtubules (CMTs) and the preprophase band, regulating onset of mitosis (Camilleri et al. 2002; Spinner et al. 2013). On the other hand, it modulates holoenzyme activity and regulates oxidative stress responses in Arabidopsis (Freytag et al. 2023; Kelemen et al. 2025a).

“C” is the catalytic subunit family. Five of these subunits were detected in Arabidopsis and rice, while in potato, there are six such isoforms (Farkas et al. 2007; País et al. 2009; Zubillaga et al. 2025). Each isoform prefers a given set of regulatory subunits: for example, C3 and C4 frequently bind to the FASS subunit (Spinner et al. 2013; Máthé et al. 2023). The Arabidopsis catalytic subunit isoforms are classified into Subfamily I (C1, C2 and C5) and Subfamily II (C3 and C4) (Farkas et al. 2007).

Although physiological roles of PP2A at the distinct subcellular levels will be discussed in detail in the sections below, their most important general functions in plants are summarized here. In the majority of the mechanisms presented below, PP2A acts as an important component of reversible protein phosphorylation (kinase-phosphatase activity balance) (Luan 2003; Máthé et al. 2023; Cortelezzi et al. 2025):

(i) They are involved in the regulation of signal transduction events related to plant hormones. A prominent example is the dephosphorylation and thereby activation of BZR1 (Brassinazole Resistant 1), an important transcription factor for the execution phase of brassinolide signaling. PP2A is also capable of dephosphorylation and thereby inactivation of BRI I, the brassinosteroid receptor, thus under certain circumstances it can inactivate signaling mediated by this plant hormone (Tang et al. 2011; Wang et al. 2016). We will discuss the involvement of PP2A in ABA and ethylene (ET) signaling in detail in the following sections.
(ii) They regulate functioning and recycling of the auxin efflux carrier family PIN, thereby influence auxin distribution within the plant (Zwiewka et al. 2019). Further stress-related details can be found in the Membranes section of this review.
(iii) They modulate the phosphorylation state, thereby regulating activities of numerous enzymes involved in key metabolic processes, a typical example being the light-dependent regulation of nitrate reductase (Creighton et al. 2017a).
(iv) They regulate the cell cycle at all three checkpoints, for example by influencing CDK activities in multiple ways (Bollen et al. 2009).
(v) They regulate plant–microbe interactions including the relationships between plants and rhizosphere bacteria.
(vi) They regulate diverse biotic and abiotic stress reactions of plants.

Since the present work focuses on abiotic stresses, we provide some general information on this issue here and show details of related subcellular events in the following sections. Evidence exists for the relevant roles of isoforms of all PP2A subunits, but the effects of different holoenzyme combinations are diverse (Han et al. 2017). Previous surveys of the involvement of PP2A in oxidative stress responses in relation to environmental challenges can be found in the reviews of Rahikainen et al. (2016) and Máthé et al. (2019). Concerning diverse “A”, “B” and “C” subunits of Arabidopsis, rice, wheat, tomato or potato, general information on their involvement in (abiotic) stress responses including drought/salt and temperature (cold/ heat) stress, can be found in the reviews of País et al. (2009), Bheri and Pandey (2019), Máthé et al. (2023) and Cortelezzi et al. (2025), among others. We show some general examples in this introductory section, then novel data/ views on subcellular mechanisms will be provided in detail in the following sections.

Transcription of genes for all catalytic subunits of PP2A is stimulated in rice leaves, while in potato leaves, this is true in case of four “C” subunits under salt stress. The transcription of three catalytic subunit isoforms (OsPP2A-2, -4 and -5) occurs at different levels in diverse organs in rice and the isoforms are also differentially expressed under combined drought-heat treatment. This organ-and isoform-dependent expression suggests that different catalytic subunit isoforms may have different functions in responses of plants to abiotic stresses (Yu et al. 2005; País, 2009). Several B” subunits contain Ca^2+^ binding EF-hand motifs and regulate calcium signaling during drought response, i.e. by mediating the dephosphorylation of VIP1, a bZIP transcription factor to promote stomatal closure (Ren et al. 2023). In potato, the expression (transcription) of all PP2A subunits studied—six of C subunit, three of A subunit and 18 of B subunit genes—is changed under drought stress or ABA treatment with mostly increasing levels during short-term salt or mannitol treatments (Zubillaga et al. 2025). The A1 subunit (RCN1) of Arabidopsis is involved in ABA-induced stomatal closure and it is important in plant responses to salt stress (Kwak et al. 2002; Blakeslee et al. 2008). ABA signaling is regulated by the B’θ subunit as well (Matre et al. 2009). Diverse catalytic subunits are involved in cold and salt stress responses in potato, tomato, rapeseed and wheat (País et al. 2009 and references therein; Zhang et al. 2021). An interesting study shows that B’γ and B’ζ subunits keep antioxidant systems at low levels which is required for normal growth under optimal environmental conditions. Double Arabidopsis mutants for these subunits show enhanced tolerance to a combination of high light intensity, heat and drought (Konert et al. 2015a).

The mechanisms of PP2A involvement in different abiotic stress responses are diverse and several aspects remain unclear. Examples related to osmotic/salt stress, where relatively more information is available (Chawla et al. 2020): (i) PP2A deactivates osmotic sensor kinase substrates; (ii) PP2A interferes with mevalonate/isoprenoid metabolic pathways involved for example in ABA biosynthesis, featuring regulatory subunits like B”α, B”β; (iii) The catalytic subunit C5 increases the activity of the vacuolar chloride channels CLC to retain Cl- in the presence of NaCl, thereby increasing salt tolerance. Other relevant mechanisms will be discussed in detail in the next sections.

It is important to mention that the activities of PP2A catalytic subunits can be affected by their post-translational modifications. Besides phosphorylation of holoenzyme subunits, for example, methylation of catalytic subunits at a C-terminal motif will change (decrease) the activity of the holoenzyme to help tolerance to heat or heavy metals. In general, methylation of the catalytic subunit is one of the requirements for binding of “B” subunits to the core enzyme (“A” + “C” subunits) (Creighton et al. 2017b, 2022). In the Membranes section of this review we will see that in relation to brassinolide (BR) signaling, there are PP2A holoenzymes where the methylation of “C” subunit is proposed not to decrease phosphatase activity. Regarding temperature stress, an early study demonstrated cold-inactivation of PP2A activities in alfalfa (Monroy et al. 1998). It later turned out that the CHIP ubiquitin ligase increases PP2A activity (the PP2A interactor is the A3 subunit) during cold treatments, but the background mechanisms remain to be established. CHIP is involved in the degradation of heat shock proteins Hsp70 and Hsp90, and its overexpression causes increased cold sensitivity in Arabidopsis (Luo et al. 2006).

PP2A interacts with BAG8 co-chaperone in the cytoplasm in tomato and this is thought to play a role in cold tolerance. However, the nature of this interaction (i.e. which PP2A subunits are involved?) and how it functions are not yet understood (Guo et al. 2024).

This review primarily discusses the subcellular localizations and functioning of diverse PP2A holoenzymes in the context of osmotic/salt and heat stress; thus, we offer a cell biology perspective of these relationships. The following sections examine different subcellular compartments. We present our view on their multifaceted functioning in the plant cell, especially in the light of novel findings in the field of protein phosphatase-abiotic stress relationships. The relevant research may contribute to the creation of stress-resistant crops by manipulating the functioning of distinct PP2A subunits. Figures and Supplementary Table 1 support our integrated view on PP2A-abiotic stress relationships.

### PP2A and abiotic stress at the subcellular level

#### Chromatin/nucleus

There are multiple effects of protein phosphatase (PP2A and/or its relative, PP1) chemical inhibitors on the organization of plant chromatin. They induce chromatin condensation, fragmentation as well as micronucleus formation that can occur in both mitotic and non-mitotic cells (Beyer et al. 2012; Máthé et al. 2021a). Concerning (stress-related) direct involvement of PP2A, there is more information on the post-translational modifications of histones.

**Fig. 1:**
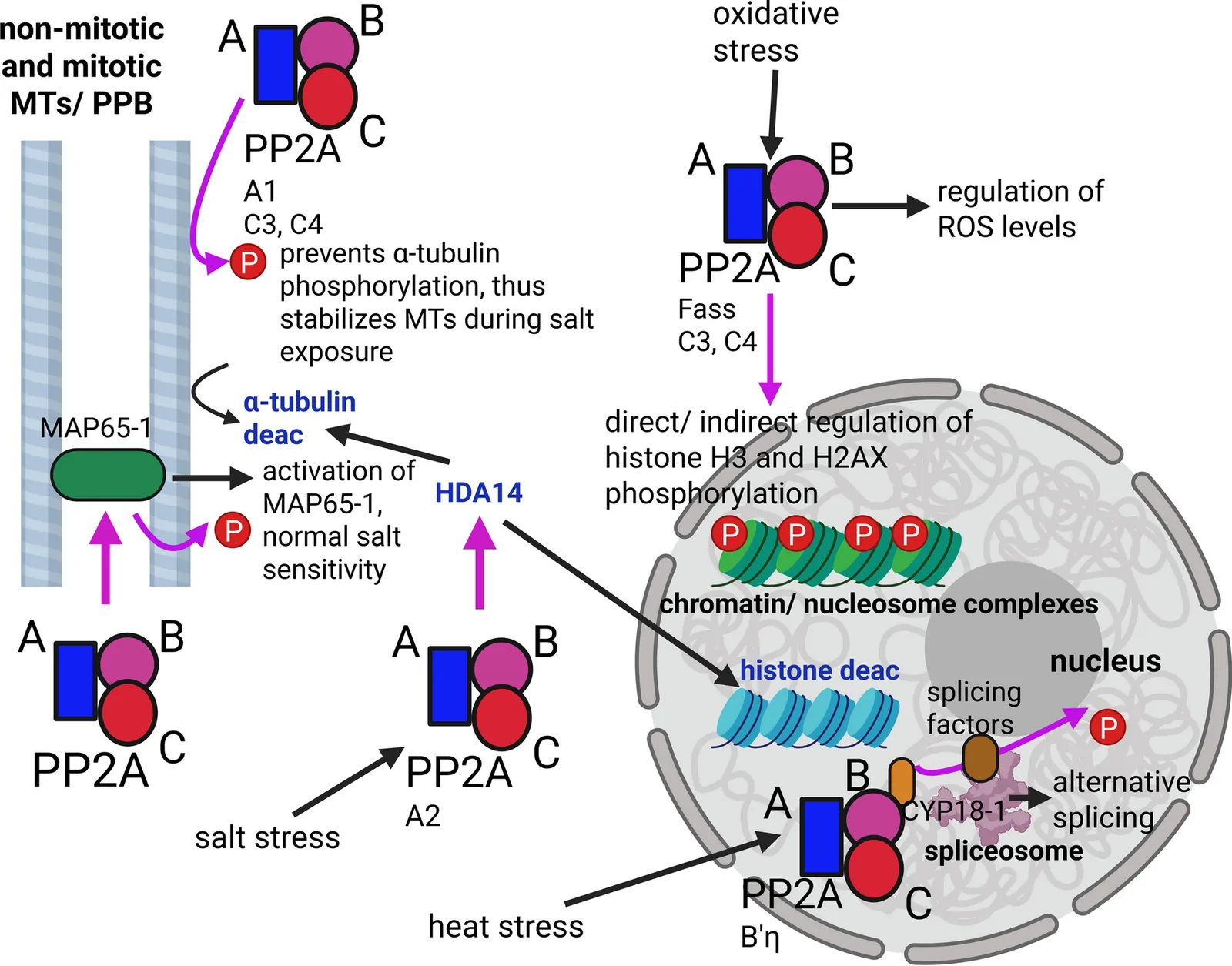
The role of PP2A holoenzymes in the regulation of oxidative/salt stress responses at the microtubule and chromatin level. HDA14 seems to be an integrator in the regulation of MT stability and histone deacetylation. Purple arrows: dephosphorylation-dependent events. Featuring PP2A subunits for given processes are indicated (where their nature is known). See the “Chromatin/nucleus” and “Cytoskeleton” sections of this review for details. Abbreviations: CYP18-1- splicing regulator; deac- deacetylation; HDA14- a histone deacetylase; MAP-microtubule associated protein; MT- microtubule; PPB- preprophase band. Figure was created with the BioRender software

Histone H3 is phosphorylated during early mitosis mainly at Ser10, Ser28 and Thr4 residues and its phosphorylation is gradually decreased during late mitosis (Houben et al. 2007). This cycle is important for the proper condensation of chromatin during early stages of cell division and for the proper segregation of sister chromatids at later stages (Houben et al. 2007; Beyer et al. 2012). Inhibitors of PP2A/PP1 induce hyperphosphorylation of histone H3, which even leads to a delay of segregation and mis-segregation of chromatids (Beyer et al. 2012). It is still debated whether PP2A or PP1 is primarily involved in dephosphorylation but recently more evidence suggests the importance of PP2A – including specific subunits such as C3, C4 and FASS (Kelemen et al. 2025a, b and Fig. 1). A member of NAPs (nucleosome assembly proteins) inhibits PP2A and the consequent dephosphorylation of histone H3 (Bíró et al. 2012). Moreover, *fass* mutants of Arabidopsis are characterized by increased reactive oxygen species (ROS) content, but decreased level of H3 phosphorylation, since FASS decreases rather than stimulates PP2A holoenzyme activity (Freytag et al. 2023; Kelemen et al. 2025a). Whether there is a direct relationship between oxidative stress and histone H3 phosphorylation in plants is an exciting future research direction; however, this observation suggests a correlation and demands more research on related molecular mechanisms (see the list of future research directions at the end of this review). It is worth mentioning that diquat (DQ), a ROS inducer, increases the phosphorylation level of histone H3 in Arabidopsis wild-type and *fass-15* mutants (Kelemen et al. 2024; Garda et al. 2025; Fig. 1). This indicates that at least the FASS subunit regulates oxidative stress responses, but the fine mechanisms are yet to be elucidated. *c3c4* and *fass-5* mutants with more severe developmental alterations than *fass-15* show decreased H3 phosphorylation levels in the presence of DQ, but here it is possible that these genotypes show general metabolic anomalies. Thus, this effect may not be related directly/specifically to PP2A; it is potentially related to a more general, stressed condition of the mutant plants (Kelemen et al. 2024). It is an interesting question whether FASS or its binding partners influence oxidative stress responses in plants exposed to drought/high salt or extreme temperatures.

Histone H2AX is a special variant of H2A. Its phosphorylated form is labeled as γH2AX, and it is considered an important inducer of DNA damage repair during oxidative stress (Fuchs et al. 2006). Specific PP2A subunits – C3, C4 and FASS—modulate its phosphorylation state as shown by the analysis of the respective mutants, but it remains to be established whether this is a direct effect of PP2A defects in the mutants, or just a non-specific reaction in the mutants due to general developmental defects and their increased sensitivity to stress (Freytag et al. 2023). Thus, it is still to be established whether PP2A is a direct modulator of H2AX phosphorylation (Fig. 1).

Interestingly, PP2A can be related to other histone post-translational modifications (PTMs) besides phosphorylation. The histone deacetylase HDA14 is of particular interest. Besides histone PTM – which may lead to gene silencing—it has a broad range of substrates at different subcellular localizations. For example, it is capable of α-tubulin deacetylation and as such, may influence microtubule (MT) stability and docking of motor proteins. HDA14 associates with the A2 subunit of PP2A and this relationship is probably not confined only to the nucleus. It was suggested that this interaction is important for a PP2A activity that regulates histone deacetylase (HDA), but further evidence is needed to determine the possible biochemical mechanism involved (Tran et al. 2012). Several members of the histone deacetylase family regulate gene expression during salt, drought and (high/low) temperature stresses (Luo et al. 2017). HDA14 mediates salt tolerance in Arabidopsis (Ueda et al. 2017). As having a wide substrate range, this protein might be important in the coordination of protein functioning via PTM at a broad range of cellular locations and may integrate the regulatory roles of PP2A at the chromatin and MT levels (Fig. 1).

We present below selected examples of the PP2A- mediated events in the nucleus that are not related to histone PTMs. Experiments performed in plants other than the Arabidopsis model show, for example, that overexpression of wheat TaPP2A-C1 catalytic subunit in tobacco confers enhanced drought tolerance and the GFP-fusion product is localized to the nucleus and cytoplasm, although the precise cytoplasmic localization (cytosol or organelles?) is not known yet (Xu et al. 2007). qPCR analysis for soybean B” subunit genes indicated *GmPP2AB”71* gene overexpression, that greatly enhanced salt/drought tolerance and the corresponding GFP-fusion product showed nuclear localization. Also, this gene product is acting through the regulation of ROS elimination to give further evidence for the PP2A-ROS metabolism relationship (Xiong et al. 2022).

In relation to heat stress, the PP2A B’η subunit interacts with CYP18-1, a splicing regulator in the Arabidopsis nucleus to dephosphorylate PRP18, a component of the spliceosomal complex. This allows proper splicing of heat-responsive pre-mRNA transcripts (Jo et al. 2022). The same research group shows more recent evidence that during heat shock at early germination, levels of B’η increase to induce alternative splicing of heat-responsive pre-mRNAs. Besides PRP18, this PP2A subunit interacted with and contributed to the dephosphorylation of other splicing factors or exon junction factors (PRP16, RH12) (Jo et al. 2025; Wang 2025).

#### Cytoskeleton

The cytoskeleton is one of the most important cellular structures in terms of structural/ functional integration of plant intracellular processes, thus has a crucial place in this review.

The organization of both microtubules (MTs) and microfilaments (MFs) is sensitive to diverse abiotic stresses (Wang et al. 2007; Kuběnová et al. 2021). For MTs, NaCl stress induces reorganization of cortical microtubules (CMTs) and alters the orientation of roots during growth (Wang et al. 2007).

The interaction between PP2A and plant cytoskeleton is relatively well understood for MTs. This refers to both mitotic and non-mitotic structures. Concerning drought/salt or extreme temperature stress, there is relatively less knowledge in the PP2A context, however we present several interesting findings below.

It has long been known that inhibitors of PP2A or those of both PP2A and its relative protein phosphatase 1 (PP1) disrupt these structures in vascular plants (Zhang et al. 1992; Ayaydin et al. 2000; Máthé et al. 2009). This is valid for different mutants of PP2A subunits as well (Camilleri et al. 2002; Spinner et al. 2013).

A prominent example for MT-PP2A interaction is the regulation of the MAP65 family of microtubule-associated proteins. AtMAP65-1 of Arabidopsis regulates bundling of mitotic MTs, and to carry out this function, a PP2A holoenzyme is needed for its dephosphorylation (Smertenko et al. 2006). This MAP is crucial for the proper MT organization – polymerization state and bundling- during salt stress and loss-of- function *map65-1* mutants show salt (NaCl) hypersensitivity (Zhou et al. 2017 and Fig. 1).

The C3 and C4 catalytic subunit isoforms are involved in the regulation of cytoskeletal organization, which applies to both CMTs and mitotic MTs. Virus-induced transient silencing of their genes delayed the recovery of Arabidopsis plants after their exposure to salt stress. Salt treatment induces the phosphorylation of α-tubulin, causing MT depolymerization and the phosphatase activity of PP2A holoenzymes containing C3 or C4 counteract this effect upon salt removal (Yoon et al. 2018 and Fig. 1). Interestingly, the interactors of these subunits are also involved in stress tolerance. The A1 subunit (RCN1) regulates PP2A activity during different abiotic, including osmotic stress conditions. This subunit has multiple subcellular localizations, including membranes, and it is involved in the PP2A-mediated regulation of polar auxin transport via PINs (Blakeslee et al. 2008 and see next sections). The *rcn1* and *c4* mutants show increased MT depolymerization and reorientation in the presence of NaCl, raising the possibility that tubulins remain hyperphosphorylated because of decreased PP2A activities in these mutants (Angelini 2014).

Besides RCN1, FASS as a B” subunit is an important interactor of C3 and C4. As part of a large protein complex (TTP, with a PP2A holoenzyme as a structural component), it localizes the holoenzyme to MTs, thereby regulating cytoskeletal organization (Spinner et al. 2013). There is increasing evidence for its importance in mediating plant stress responses. Its loss-of-function mutants show increased ROS content and stimulation of Cu/Zn superoxide dismutase activity in Arabidopsis seedlings (Freytag et al. 2023). Moreover, as we have shown in the previous subchapter, FASS may influence oxidative stress responses at the level of histone H2AX as well. Therefore, this regulatory subunit may regulate such responses at multiple levels. Such findings indicate that FASS-containing PP2A holoenzymes may function outside the cytoskeleton as well.

As we have shown in the previous subchapter, the phosphorylation of histone H2AX or H3 is or may be regulated by PP2A including C3, C4 and FASS subunits. On the other hand, as we see here, these subunits regulate CMT and mitotic MT organization. Is there a coordination between PP2A-mediated chromatin and cytoskeleton regulation? Sister chromatid segregation is regulated by this phosphatase (and probably by PP1) at the level of both histone H3 phosphorylation and the organization of mitotic spindle at the level of spindle checkpoint of cell cycle. These two events seem not to be correlated, however, disorders for both of them induce alterations on chromatid segregation and arrests of mitosis in broad bean (*Vicia faba*) (Beyer et al. 2012). In Arabidopsis, *fass* mutants show mitotic arrests, but these are not associated with concerted alterations in spindle MT organization and histone H3 phosphorylation (Kelemen et al. 2025a). All these findings show that specific PP2A subunits such as FASS and C3/C4 regulate both mitotic MTs organization and histone phosphorylation. In this context, even though the above functions are exerted via different pathways (their molecular mechanisms should be further elucidated), these specific subunits are involved in multiple regulatory events.

Concerning MFs, a study has shown that the PP2A/PP1 inhibitor microcystin-LR (MCY-LR) induces actin reorientation and depolymerization in rice roots, however the exact mechanisms are not known (Pappas et al. 2020). MFs are also known to be crucial for salt tolerance in Arabidopsis, meanwhile high NaCl concentrations decrease the stability of actin filaments (Wang et al. 2010). MF organization is affected by ROS (Majumdar and Kar 2020). It remains to be established whether PP2A plays any roles in these events. In this review we do not concentrate on light (e.g. high light intensity)—mediated stresses; nevertheless, it is interesting to see, that ADF/cofilin phosphorylation assays revealed: the C2 subunit of PP2A regulates/activates this actin-binding protein, to mediate depolymerization and rearrangement of microfilaments during high-fluence blue light treatments and in consequence, promotes avoidance movement of chloroplasts in Arabidopsis (Wen et al. 2012). An exciting question is: what about the PP2A-actin binding protein relationship under other abiotic stresses?

### Stress-related PP2A functioning at the membrane level and related cytosolic events

The wheat B”α subunit is localized in the plasma membrane, cytoplasm and nucleus, expressed mainly in roots and maintains root (primary and lateral) growth under different stress treatments (NaCl, mannitol/PEG). Its expression (transcript) levels were increased at different stress exposures (NaCl, PEG, cold, ABA). B”α interacted with an “A” subunit related to Arabidopsis A1/RCN 1 and to a “C” subunit related to Arabidopsis C3 and C4 (Liu et al. 2014). This is an example for the possible involvement of membrane-localized PP2A in abiotic stress responses.

**Fig. 2:**
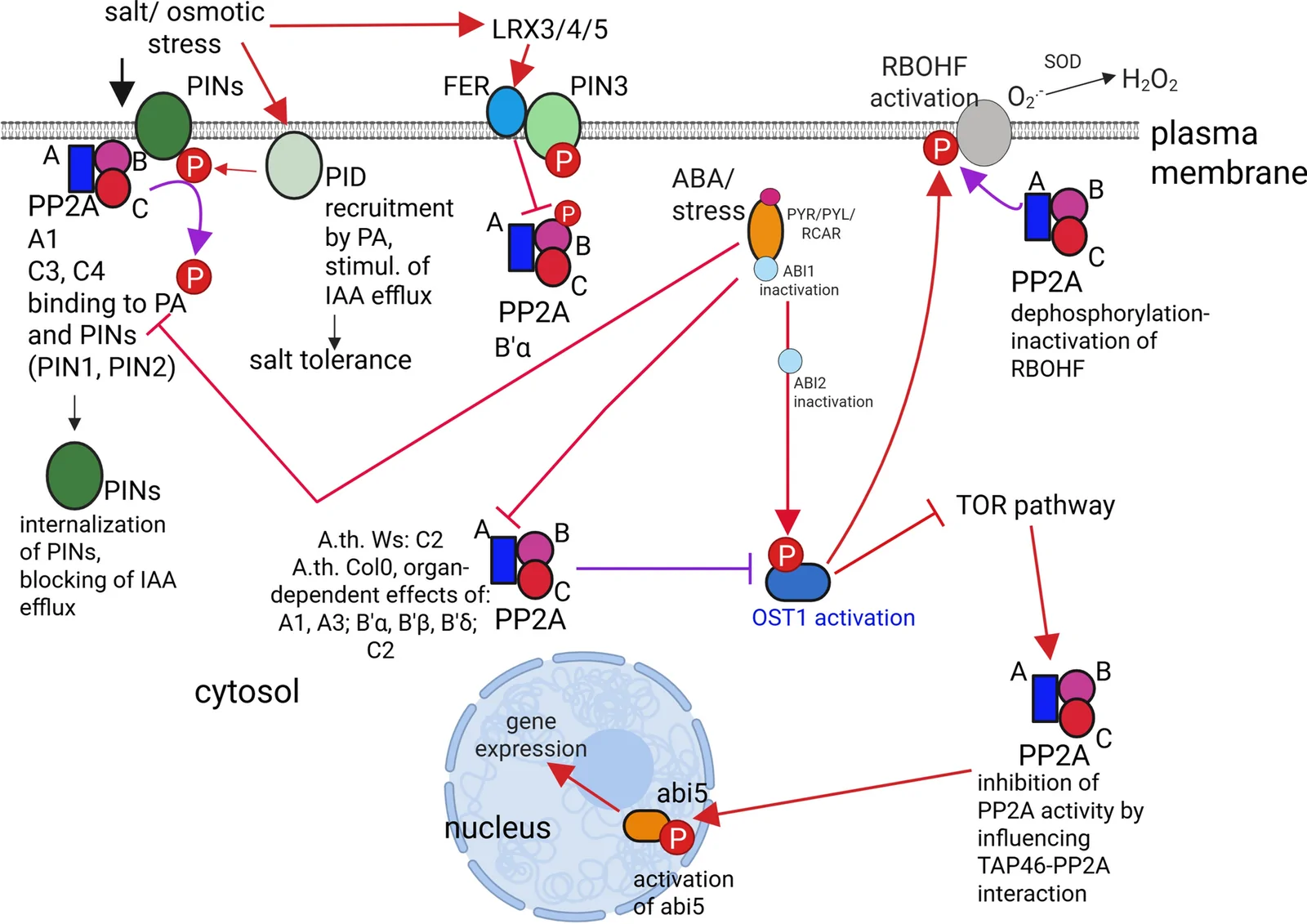
The role of PP2A holoenzymes in the regulation of ABA/salt/osmotic stress responses at the plasma membrane and cytosolic levels. Red arrows/ inhibition pathways: phosphorylation dependent events, purple arrows/inhibition pathways: dephosphorylation dependent events. Featuring PP2A subunits for given processes are indicated (where their nature is known). See the “Membranes” section of this review for details. Abbreviations: ABI1- a protein phosphatase PP2C; A.th. – *Arabidopsis thaliana*; FER- FERONIA kinase; LRX3/4/5 – cell wall leucine-rich extensin; OST1- OPEN STOMATA SnRK2 kinase; PA- phosphatidic acid; PID- PINOID kinase; PYR/PYL/RCAR- ABA receptor; RBOHF- Respiratory Burst Oxidase Homologue; TAP46- type 2A phosphatase associated protein 46 kDa; TOR- Target of Rapamycin; Ws – (ecotype) Wasillewskiya. Figure was created with the BioRender software

As we have mentioned, RCN1, an A1 subunit important for plant development is localized among other cell structures at membranes. This refers especially to the plasma membrane and the microsomal fraction of root cortical cells, where A3 can also be localized. Since these subunits do not have membrane-binding domains, this interaction is probably indirect, via typical membrane proteins (Blakeslee et al. 2008). Such proteins are for example a plasma membrane ATPase as well as the auxin efflux carriers PIN1 and PIN2 (Fuglsang et al. 2006; Michniewicz et al. 2007). The A1 subunit binds PP2A holoenzyme (indirectly) to plasma membrane phosphatidic acid to regulate the phosphorylation state, therefore membrane localization of PIN1 (Gao et al. 2013). Regarding stress responses, RCN1 clearly contributes to osmotic stress/salt tolerance as demonstrated by the increased sensitivity of loss-of function mutants to these abiotic stresses (Blakeslee et al. 2008). Figure 2 shows several aspects of A1-membrane interactions.

Comprehensive descriptions are available on the PP2A-mediated regulation of PIN functioning, therefore of polar auxin transport (Zwiewka et al. 2019; Máthé et al. 2021b). In brief, PP2A dephosphorylates PINs and at least for PIN1 and PIN2, this PTM allows recycling of these efflux carriers via internalization by vesicle transport, enabling them to enter a new IAA efflux cycle. The phosphorylation state of PINs also influences their auxin transport activity. Besides A1, the catalytic subunits C3 and C4 are thought to be the main PP2A components that participate in this process (Ballesteros et al. 2013). Since FASS is a known interactor of both A1 and C3/C4, it is likely involved in PIN regulation as well. According to the model proposed by Han et al. (2017 and see left side of Fig. 2), salt stress redistributes phosphatidic acid (PA) in the plasma membrane to the cell side closest to high external salt concentration, which stimulates stable internalization of PINs (their delocalization from the plasma membrane), therefore inhibits further auxin efflux. The high local amount of PA binds the PP2A complexes stably to the plasma membrane via RCN1, and decreases activity of the holoenzyme, leading to disruption of PIN recycling. This induces auxin redistribution leading to avoidance movement of roots that protects them against high local salt concentrations. Furthermore, a more recent study shows in addition, that PINOID (PID) kinase—which functions oppositely to PP2A and thus phosphorylates PINs—is also recruited to the plasma membrane by phosphatidic acid. During the induction of salt tolerance in Arabidopsis, besides PP2A, increased phospholipase D (PLD) activity induces the local accumulation of PA, which recruits more PID kinase and increases its activity. This will redistribute auxin and maintains root growth during salt stress (Wang et al. 2019 and Fig. 2). An interesting recent study shows that cell wall leucine-rich extensin (LRX3/4/5) stimulates the activity of plasma membrane FERONIA, a receptor-like kinase 1-like (RLK1L) protein that phosphorylates PIN3, while it inhibits PP2A activity via phosphorylating the Bα subunit. This mechanism regulates auxin transport and increases tolerance during salt stress (Liu et al. 2026; Fig. 2).

To summarize the regulation of PINs, their reversible protein phosphorylation influences auxin distribution in roots under abiotic stresses and specific PP2A subunits, featuring A1 and C3/C4, are crucial players here.

ABA, known for its role in the regulation of stress responses, is involved in PP2A-regulated PIN functioning at several levels. The classical view of the ABA signaling pathway: it binds to the soluble receptor PYR/PYL/RCAR, which inhibits PP2C (ABI1, a metal-dependent phosphatase distinct to the PP2A holoenzyme family), increasing the phosphorylation state of several signaling intermediates (see Zhu 2016 for a review). There is increased evidence for the involvement of PP2A in ABA signaling and more recently, it was shown that binding of ABA to its receptor during salt/osmotic stress inhibits PP2A activity as well. C3 and C4 are the inhibited catalytic subunits, thus, they are not be able to dephosphorylate PINs (Fig. 2). As a consequence, auxin transport and recycling is modified to change root architecture in relation to stress adaptation (Li et al. 2020).

Many PP2A subunits, including those playing roles in abiotic stress responses, are localized in the cytosol as well (see for example: Máthé et al. 2021b, 2023; Zubillaga et al. 2025). One such subunit is B’γ (Konert et al. 2015b), but as we will see below, several other subunits are also functioning in the cytosol in relation to stress reactions. A key event in this sense is ABA signaling. The result of PP2C inhibition is that OST1 (OPEN STOMATA 1), a cytosolic/nuclear SnRK2 (Sucrose Nonfermenting1-related protein kinase 2) remains phosphorylated, thus active, and will regulate transcription factors in the nucleus as well as open anion-out channels to promote stomatal closure (Mittler and Blumwald 2015; Gahir et al. 2020). PP2A is also capable of regulating OST1 in an ABA-dependent manner. This interaction occurs at two levels (Fig. 2). Firstly: OST1 phosphorylates the respiratory burst oxidase homologue RBOHF, a major ROS generator at the plasma membrane and PP2A interferes with this process probably by directly dephosphorylating RBOHF (Rahikainen et al. 2016; Máthé et al. 2019). Secondly: there is a direct interaction between OST1 and PP2A. Arabidopsis (ecotype Ws) mutants for the catalytic subunit C2 show increased ABA sensitivity, probably because OST1 remains active here (Pernas et al. 2007). The work of Waadt et al. (2015) gives more details on OST1-PP2A interactions. Interestingly, single PP2AC2 mutants of the Col0 ecotype of Arabidopsis were not more sensitive to ABA, in contrast to the previous observations on Ws. Meanwhile, single A1 or double A1-C5 and C3-C5 mutations showed decreased sensitivity to ABA during seed germination or stomatal closure, but increased sensitivity in terms of root growth. These contrasting results suggest that the type of biochemical interaction between OST1 and PP2A in the presence of ABA depends on the subunit composition of PP2A holoenzyme, on the organ/tissue type as well as on genotype. In addition, as we have shown above, ABA may interfere with PP2A at other levels with other mechanisms such as regulation of polar auxin transport. Waadt et al. (2015) predict that the primary interaction partners with OST1 are the “A” subunits and through these, a number of “B” and “C” subunits participate in this interaction. According to BiFC (bimolecular fluorescence complementation) assays, the interactions between OST1 and diverse PP2A holoenzymes are located mainly to the nucleus, but are also localized to the cytosol/cytoplasm. Since strong interactions between diverse PP2A subunits and OST1 are observed at the level of cytosol and distinct cytoplasmic structures, we can presume that at least part of the OST1/PP2A interaction occurs at this level. Main interactors with OST1 were A1, A3, B’α, B’β and B’δ (Waadt et al. 2015). All the above findings on OST1-PP2A interactions –see Fig. 2- raise many questions about the underlying molecular mechanisms that need further investigation.

ABA signaling does have connections to the Target of Rapamycin (TOR) pathway as well (Fig. 2). TIP41 and TAP46 proteins are PP2A regulatory subunits and components of this pathway. TAP46 is phosphorylated by TOR and modulates PP2A activity under non-stress conditions (Ahn et al. 2011). In wheat, TIP41 and TAP46 interact with the catalytic subunit TaPP2A-2 and by inhibiting its activity, they promote drought tolerance. TaTIP41 localizes not only in the nucleus, but in the cytoplasm as well. The TIP41-TAP46 interaction seems to be universal in plants and all Arabidopsis PP2A subunits are predicted to interact with TIP41 (Punzo et al. 2018a). Moreover, TIP41 is localized in the nucleus and cytoplasm in Arabidopsis, as in wheat (Punzo et al. 2018b).

The TOR pathway is functional not only under optimal conditions, but at different stress exposures as well (Ma et al. 2023). TAP46, a regulatory subunit of PP2A is also related to ABA signaling in relation to its interaction with TIP41 and inhibition of PP2A: it prevents dephosphorylation of ABI5 transcription factor, which may be important in the stress tolerance promoting activity of ABA (Punzo et al. 2018b; Fu et al. 2020; Fig. 2). Other works show that ABA inhibits the TOR pathway by activating SnRK2 (OST1?), which in turn inhibits TOR kinase activity (Saksena et al. 2020). As we have shown above, this implies the inhibition of PP2A activity to maintain SnRK2 functionality (Fig. 2). We mention here that TAP46 associates with PP2A, and its transcript level increases at chilling in Arabidopsis (Harris et al. 1999). An exciting future research direction is further mapping of PP2A subunits related to the connection of TOR pathway to ABA signaling and to explore the related consequences for plant stress resistance/creating of resistant cultivars.

Taken together, these studies clearly show that ABA/stress signaling involves PP2A holoenzymes at different subcellular locations. Thus, these phosphatases seem to integrate ABA-mediated responses in the whole cell. Details on the different ways for PP2A functioning in relation of ABA signaling can be seen on Fig. 2.

**Fig. 3:**
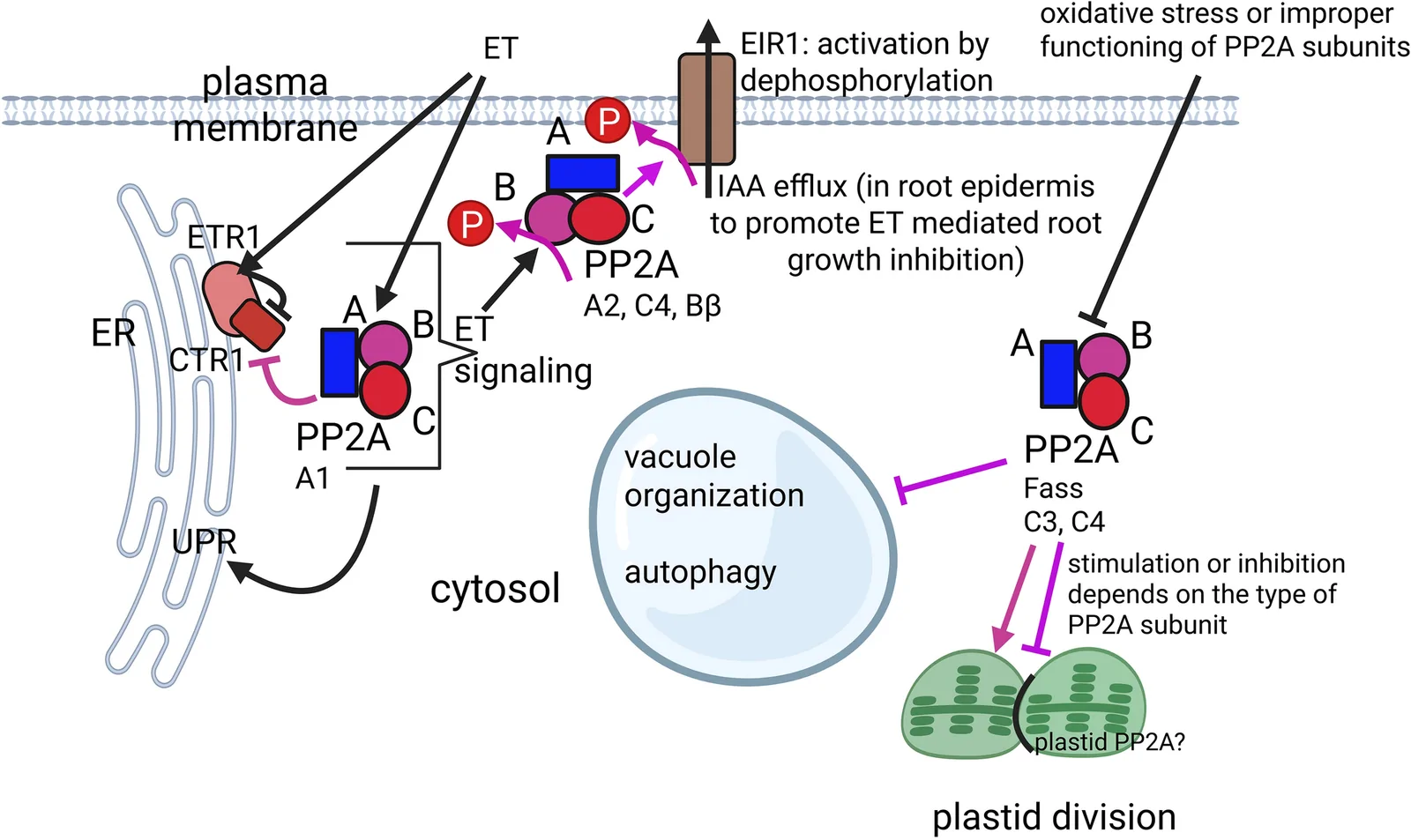
The role of PP2A holoenzymes in the regulation of ethylene/abiotic/oxidative stress responses at the plasma membrane and membrane compartment levels. For ethylene (ET) signaling, relations to auxin transport are shown as well. Although a holoenzyme with A1 subunit is involved in ET signaling as well as the UPR, it is not proven that ET signaling is connected to UPR in the PP2A context. Regarding plastid division, we propose that besides “host cell” holoenzyme, a plastidial PP2A activity also regulates this process. Featuring PP2A subunits for given processes are indicated (where their nature is known). Purple arrows/inhibition pathways: dephosphorylation dependent events. See the “Membranes” and “Plastids and mitochondria” sections of this review for details. Abbreviations: CTR1- a negative regulator of ethylene (ET) response; EIR1- an auxin efflux carrier; ER- endoplasmic reticulum; ETR1- ET receptor; UPR- unfolded protein response. Figure was created with the BioRender software

Besides the cytoskeleton, the structural organization of the endoplasmic reticulum (ER) is crucial for the integrated functioning of a eukaryotic cell (Mathur et al. 2022). In mice, the association of the anti-apoptotic protein Bcl2 and PP2A is necessary for maintaining the stability of this anti-apoptotic protein and protects cells from ER stress (Lin et al. 2006). For plants, two studies are of particular interest in terms of ER stress/unfolded protein response (UPR). *rcn1* (A1 subunit) mutants of Arabidopsis were less susceptible to the tunicamycin-induced ER stress (according to the expression levels of UPR-related genes, e.g. crt1 for calreticulin), and this was accompanied by reduced PP2A activity of mutant seedlings. Cantharidin, a PP2A inhibitor, decreased UPR in the mutants and wild-type plants as well. These findings show that PP2A activity is necessary for UPR, which is essential for stress defense (Ko et al. 2023 and Fig. 3). An earlier study showed that the B’γ subunit is required for proper PP2A activity to dephosphorylate and thus activate calreticulin 1 during the plant immune response (Trotta et al. 2011). Whether drought/salt or heat stress influences UPR in relation to PP2A, remains an open question.

Is there a connection between ABA signaling, PP2A and ER stress? Although ABA induces the expression of genes related to ER stress (Wang et al., 2013), the involvement of PP2A has not yet been demonstrated.

From the above studies in becomes clear that PP2A subunits, featuring A1 regulate stress-related ABA signaling (Fig. 2).

MCY-LR inhibits PP2A in Arabidopsis and it induces clustering of plastids around the ER membranes around the nucleus (Máthé et al. 2024). This may be related to the role of PP2A in regulating proper functioning of membrane contact sites between the ER and plastids. An interesting future research question would be: is there such a relationship under abiotic stress conditions?

A prominent example for PP2A-membrane compartment relationships is the signaling pathway(s) for ethylene, a well-known stress-related plant hormone. Ethylene (ET) receptors like ETR1 are localized to the ER membrane and bind CTR1, a negative regulator of ET signaling. According to a model of Yang et al. (2013), in the presence of ET, a PP2A holoenzyme that contains A1 (RCN1) subunit contributes to the inactivation of CTR1, thus to the activation of the signaling pathway. Another level of ET signaling-PP2A interaction involves a holoenzyme containing A2, C4 and Bβ subunits. In the presence of ET, Bβ is dephosphorylated that activates holoenzyme to dephosphorylate the auxin efflux carrier EIR1, which promotes auxin transport in the epidermis of the root elongation zone and changes auxin distribution to induce the ET mediated inhibition of root growth (Shao et al. 2022). See Fig. 3 for the ET-PP2A relationship. It is clear that besides ABA (see previous sections of this subchapter), PP2A-regulated ET signaling clearly influences tissular auxin distribution during stress.

In terms of other, hormone-related signaling pathways, brassinolide (BR) is also playing in the responses of plants to different abiotic stresses. We have already discussed in a previous review the role of PP2A subunits of the B’η subfamily in the inactivation of BRI1-BAK1 receptor-coreceptor complex at the level of plasma membrane (Máthé et al. 2023). An interesting model on BR signaling-PP2A relationship was suggested by Wu et al. (2011). According to this, BR signaling induces the production/activation of a leucine carboxymethyltransferase (LCMT) enzyme, which methylates a PP2A-C subunit that will be transported from the plasma membrane to the endosomes that contain BRI1 (BR receptor). Here, PP2A will dephosphorylate BRI1 which will then be degraded, possibly in the vacuoles. This model is partly hypothetical and it does not show which particular PP2A subunits are involved.

Wheat TaPP2AbB”-γ is a regulatory subunit that is proposed to interact in the cytosol with wheat BZR1, a transcription factor related to BR signaling. BZR1 is activated upon dephosphorylation and enters into the nucleus to induce genes related to diverse abiotic stresses (NaCl, osmotic, cold). TaPP2AbB”-γ is related to FASS subunit of Arabidopsis, but more mechanistic studies are needed to clarify the above stress relationship (Liu et al. 2019). This subunit is localized in the cytosol and nucleus of wheat protoplasts (Liu et al. 2019).

Returning to the ER-PP2A relationship: emerging evidence shows the importance of ER-cytoskeleton relationship in the intracellular dynamics of plants (Schattat et al. 2011; Mathur et al. 2022). However, there is still a lack of knowledge in terms of the relevant role of PP2A for both optimal and stressed conditions.

MCY-LR inhibits PP2A activity in Arabidopsis hypocotyls and induces vacuole fragmentation at short-term inhibitor treatments as well as autophagy at long-term exposures (Nagy et al. 2018; Máthé et al. 2024). Moreover, clustering of tonoplast-coated vesicles and vacuolar fragmentation occurs in hypocotyl cells of Arabidopsis *c3c4* and *fass* mutants as well (Juhász et al., 2023). These findings indicate the involvement of PP2A in vacuolar organization. Based on the analysis of diverse knockout mutants, Hu et al. (2017) found that C5 catalytic subunit maintains root and shoot development at reasonable levels during salt exposure (75–100 mM NaCl), but not mannitol exposure. Moreover, as we mentioned in the Introduction, C5 interacts with four tonoplast chloride channel proteins (CLCs), but it is not known whether this interaction occurs at the tonoplast or during prior vesicle traffic events related to vacuole biogenesis. It seems this interaction is independent to ABA signaling that also influences chloride channels. Thus, many issues are still to be established related to PP2A-abiotic stress-vacuole relationships.

### The plastid system and mitochondria

In relation to organelles with their own, prokaryotic-type genomes, there is less knowledge regarding stress-related involvement of PP2A. MCY-LR has a stimulatory effect on chloroplast division in Arabidopsis hypocotyls, but amplifies the inhibitory effect of methyl viologen (MV), a ROS inducer (Máthé et al. 2024). This indicates the involvement of PP2A in the regulation of plastid division and a relationship between phosphatases and oxidative stress. For the latter, optimal PP2A activity has somehow a protective effect (Fig. 3). On the other hand, we observed an inhibition of chloroplast division in the small-sized chloroplasts (longitudinal diameter ≤ 6 µm) of the *c3c4* mutant, which indicates that although the total PP2A subunit pool has a modulatory effect, the C3 and C4 subunits are important for maintaining the normal level of chloroplast division (Máthé et al. 2024). Mechanistic details for these phenomena remain to be established.

There is a debate as to whether plastids have their own true PP2A (Sun and Markwell 1992; Pujol et al. 2000). A later study shows that MCY-LR inhibits PP2A (-like) and PP1 (-like) activity in relation to plastid division in isolated chloroplasts of Arabidopsis hypocotyls (Máthé et al. 2024). PP2A is inhibited in whole hypocotyl extracts as well, and it was proposed that chloroplast division is related to both plastidic and whole-cell phosphatases (Máthé et al. 2024). Unraveling the mechanisms of plastid division as regulated by PP2A under optimal and stress conditions is a matter of future research. It is worth mentioning that the formation of stromules, plastid stroma extensions having a role in establishing plastid-ER connections, is slightly influenced by MCY-LR (Juhász et al., 2023).

Although it is not strictly related to this review, interesting studies show the involvement of PP2A in the regulation of blue light-induced chloroplast avoidance movement (see the Cytoskeleton section of this review).

There is less knowledge on PP2A-stress issues in mitochondria. MCY-LR disturbs the normal fission–fusion cycle of mitochondria to increase the occurrence of fissioned organelles, which indicates that PP2A modulates the fission process (Juhász et al., 2023). The B’γ subunits interact with aconitase (ACO3) to activate mitochondrial alternative oxidases and this is related to the tolerance against different abiotic stresses (e.g. UV-B). This ACO3-PP2A interaction occurs at the cytosolic and not the mitochondrial level (Konert et al. 2015b; Pascual et al. 2021). Currently we do not know about such interactions during heat or drought/osmotic/salt stress.

## Overview and future research directions

As we attempted to show in this review, the involvement of PP2A holoenzymes and their particular subunits in relation to salt/drought and extreme temperature stress in plants is complex and occurs at multiple cellular levels. PP2A plays important roles at different subcellular locations in the signaling pathways of stress-related plant hormones. Among them, we focus mainly on abscisic acid and ethylene, where recent research provides new data on PP2A that contribute to a better understanding of these signal transduction events.

Within the holoenzyme, many types of “B” subunits are involved in the modulation of protein dephosphorylation at multiple subcellular locations and for multiple stresses. We show that PP2A may function as one of the intracellular integrators of different subcellular events during stress responses in plants. In addition, recent research shows that specific subunits are particularly important in the localization and stress-related functioning of holoenzymes: we highlight here the A1, C3-C4 and possibly, FASS subunits, with localizations at membranes and the cytoskeleton. In addition, C3 and C4 may be involved in the regulation of plastid division and FASS, in the organization of chromatin. Thus, the above PP2A subunits may function as specific integrators of abiotic stress responses in the whole cell, as the Figures in this review show as well.

This review shows that PP2A connects apparently distinct cellular events. A prominent case is that of ABA signaling as a modulator of polar auxin transport via inhibition of PP2A functioning (Fig. 2). On the other hand, PP2A holoenzymes are good candidates for regulating multiple stress responses, though in many cases these are unrelated events driven by different holoenzyme combinations. We give ABA again as an example: it regulates both apoplastic ROS formation and auxin redistribution in a PP2A-dependent manner, but these two pathways are independent (Fig. 2). Besides Figures, Supplementary Table 1 shows the subunits (their locations and functions) most relevant for the PP2A-drought/salt/temperature stress relationships.

Increasing evidence shows that in eukaryotes, including plants, the concerted functioning of the cytoskeleton and the ER including the regulation of intracellular dynamics, is crucial for the integrated functioning of the cell. It will be a great research challenge to determine the involvement of PP2A subunits in the maintenance of cell functions under abiotic stresses, in relation to this cytoskeleton-ER interaction. Relevant research questions would be whether PP2A regulates the cytoskeleton-ER relationship in plant cells? What is happening during exposure to stresses?

The study of PP2A – subcellular biochemical/cell physiology events—abiotic stress relationships may also have practical applications: a better understanding of the relevant functions of PP2A subunits will help manipulate their expressions/activities to obtain resilient crops. In addition to the above major challenge, other important future research directions/questions (most of them already mentioned in the previous sections) include the following:

1. Does FASS regulate oxidative stress responses during drought/ salt or heat/cold treatments?
2. Is there a direct relationship between histone H3 phosphorylation and ROS production, and how is PP2A involved in this?
3. If FASS modulates histone phosphorylation directly, this means that it can be localized in the nucleus as well. This must be proven.
4. Does PP2A regulate microfilament organization during abiotic stresses and is this mediated through activation/inactivation of actin-binding proteins?
5. Is FASS involved in the regulation of PIN functioning and recycling?
6. What is the molecular mechanism of OST1-PP2A interaction during ABA signaling?
7. Besides TAP46, what PP2A subunits link ABA signaling to the TOR pathway?
8. Does PP2A regulate UPR under drought/ salt/ heat stresses?
9. Does PP2A regulate the structural integrity of the ER and the functioning of its membrane contact sites with diverse organelles during abiotic stress?
10. How does PP2A regulate vacuole organization and tonoplast functioning during abiotic stress tolerance?
11. How does PP2A regulate plastid division under drought/salt stress?
12. What is the involvement of PP2A in cold stress tolerance?

## Supplementary Information

Below is the link to the electronic supplementary material.

Supplementary file1 (PDF 134 KB) 

## Data Availability

All findings presented in the present study are available within the references cited in this review paper.
