# Supplementary material for: A current view on the involvement of protein phosphatase PP2A subunits in the responses of plants to abiotic stress at the subcellular level
Source: Plant Cell Rep. 2026 Aug 20;45(9):250. doi: 10.1007/s00299-026-03931-5 (PMC13490214; doi:10.1007/s00299-026-03931-5)
Supplement: Supplementary file 1 [file 299_2026_3931_MOESM1_ESM.pdf]

Supplementary Table 1. A survey of PP2A subunits relevant to this review. Question marks refer to proposed, but not fully proven localizations.

**Subunit**	**Plant**	**Subcellular localizations**	**Functions in relation to drought/salt/ temperature stress**	**References**
**Scaffolding (A) subunits**				
A1 (RCN1)	*A. th.*, *T. ae.*	nucleus, MTs, plasma membrane, ER membrane?, cytosol/cyto-plasm	• ABA-induced stomatal closure, regulation of ABA signaling at the plasma membrane and cytosolic levels<br>• Responses to salt stress at multiple levels (e.g. plasma membrane, MTs) including regulation of PIN phosphorylation/ tissular auxin distribution<br>• Promotion of ET signaling<br>• Promotion of the UPR	Blakeslee et al., 2008; Yang et al., 2013; Waadt et al., 2015; Ko et al., 2023; Liu et al., 2026
A2	*A. th.*	nucleus, plasma membrane?, cytosol	• Necessary for HDA14 activity to regulate drought, salt and temperature stress<br>• ET mediated activation of the auxin efflux carrier EIR1	Tran et al., 2012; Ueda et al., 2017; Shao et al., 2022
A3	*A. th.*	cytosol, membranes	• Regulation of ABA signaling as an interactor of OST1	Blakeslee et al., 2008; Waadt et al., 2015
**Regulatory (B) subunits**				
Bα	*A. th.*	plasma membrane?	• Interacts with FERONIA kinase to maintain phosphorylated state of PIN3, which regulates auxin transport under salt/ osmotic stress	Liu et al., 2026
Bβ	*A. th.*	plasma membrane?	• During ET signaling, this subunit activates PP2A to promote auxin transport by EIR1 under stress	Shao et al., 2022
B'α, B'β	*A. th.*	nucleus, cytosol	• Interact with OST1: PP2A is inhibited during ABA signaling in an organ-dependent manner to maintain OST1 in an activated (phosphorylated) state	Waadt et al., 2015
Members of the B'η subfamily (B'γ, B'η)	*A. th.*	nucleus, cytosol	• B' η contributes to the dephosphorylation of PRP18 to promote splicing of heat-responsive pre-mRNA transcripts<br>• Besides B'α, B'β, B'γ is an interactor of OST1	Konert et al., 2015b; Jo et al., 2022, 2025

B"α	*T. ae.*	plasma membrane, nucleus, cytoplasm	• Expressed mainly in roots; regulates root growth under osmotic and salt stress.	Liu et al., 2014
B"71	*G. max*	nucleus	• Contributes to ROS elimination during drought/salt stress	Xiong et al., 2022
B" FASS (TON2) and related TaPP2AbB"-γ	*A. th.*, *T. ae.*	nucleus?, MTs, cytosol, membranes?	• Regulates the phosphorylation of histone H2AX and H3 and of other, yet unknown substrates during oxidative stress.<br>• Regulates cellular ROS content and Cu/Zn-SOD activity<br>• The wheat homologue regulates osmotic/salt and cold stress responses.	Spinner et al., 2013; Liu et al., 2019; Freytag et al., 2023; Máthé et al., 2023; Garda et al., 2025; Kelemen et al., 2025a
TIP41/TAP46	*T. ae.*	nucleus, cytoplasm	• Involved in the TOR pathway, they inhibit PP2A activity during ABA signaling.	Ahn et al., 2011; Punzo 2018; Ma et el., 2023
**Catalytic (C) subunits**				
Subfamily I: C2	*A. th.*	cytosol	• Regulates ABA signaling in an organ-dependent manner (see information for the interactors A1, A3 and B'α, B'β above, in this Table).	Waadt et al., 2015
Subfamily II: C3, C4	*A. th.*, *T. ae.*	nucleus, MTs, plasma membrane, cytosol	• Regulates histone H3and H2AX dephosphorylation during oxidative stress (together with FASS, see above).<br>• Dephosphorylate α-tubulin to re-build MTs after recovery from salt stress.<br>• Dephosphorylate PINs during salt stress to redistribute auxin. During ABA signaling, they may be inhibited to block PIN recycling (A1-C3/C4 complexes are affected, see above).	Spinner et al., 2013; Yoon et al., 2018; Li et al., 2020; Shao et al., 2022; Liu et al., 2026

Abbreviations: *A.th.- Arabidopsis thaliana*; ET- ethylene; *G. max - Glycine max*, soybean; HDA14- histone deacetylase 14; MT- microtubule, OST1- Open Stomata 1; ROS- reactive oxygen species; *T. ae.- Triticum aestivum*, wheat; TOR – Target of Rapamycin; UPR- unfolded protein response
